# Supplementary material for: Stoichiometric characteristics of woody plant leaves and responses to climate and soil factors in China
Source: PLoS One. 2023 Sep 21;18(9):e0291957. doi: 10.1371/journal.pone.0291957 (PMC10513206; doi:10.1371/journal.pone.0291957)
Supplement: Attachment — Submitted filename: Reply to the editor and reviewers20230525.doc [file pone.0291957.s003.doc]

#### **Reply to the editor and reviewers**

#### **Manuscript ID**: PONE-D-23-11129

**Title:**	Stoichiometric characteristics of woody plant leaves and responses to climate and soil factors in China

**Authors:** Xiangguang Duan

Dear Ph.D. Guo,

First of all, we should thank you and the reviewers for your expert comments and valuable suggestions to our manuscript. After reading the comments, we came to realize that there were indeed some weak points and unsuitable expression in our original manuscript. The issues raised by you and the two reviewers were of great help for our revision.

In the past few weeks, we have seriously revised the original manuscript. As you will see, all the questions and suggestions were taken into consideration in the revised manuscript. Below we explain in detail how the questions and suggestions were addressed.

#### **Reply to the reviewer 1**

Reviewer #1: Review summary:

The research topic appears to be of interest and appropriate for publication in PLOS ONE (ISSN 1932-6203). This manuscript examines the content of "Stoichiometric characteristics of woody plant leaves and responses to climate and soil factors in China" (PONE-D-23-11129), and reports the data sets on leaf nitrogen and phosphorus concentrations and N: P ratios in Chinese woody plants have been compiled and classified by life forms.

There are some technical and structural issues in this study that need to be addressed during the revision process.

**Question 1:** Why is there a discrepancy between the author affiliation provided in the journal system and the title page? Regarding product ownership, it is a serious issue

**Reply:**

- Thank you for your comments. Due to applying to this journal during my doctoral studies at Beijing Forestry University, the information was not updated in a timely manner and has now been revised. My current employer is Xinyang Normal University.

**Question 2:** L8: Language quality can be improved here (e.g., ecological stoichiometry) as well as similar minor errors in each section.

**Reply:**

- Thank you for your comments. It has been modified by language polishing company according to your suggestions.

**Question 3:** L23: The standard deviation should be added to these mean values as means + SD

**Reply:**

- Thank you for your comments. The standard deviation had been added to these mean values as means + SD (20.77±8.12 mg g^-1^ and 1.58±1.00 mg g^-1^).

**Question 4:** L117-124: There is no clear explanation of the research hypothesis in this study.

**Reply:**

- Thank you for your comments. It has been modified in the manuscript according to your suggestions. The main purpose of this study is to test the universality of leaf N and P chemometric relationships and the influencing factors of N-P scaling exponent in Chinese woody plant.

**Question 5:** L117-124: Aims should be categorized into points that correspond to the headings of results and discussion in order to clarify them more clearly.

**Reply:** Thank you for your comments. It has been modified in the manuscript according to your suggestions. In this paper, the overall chemometrics of leaf N and P are discussed first, as they can be used as a basis for comparison with other studies. Next, the N and P contents and scale indices of different life forms were determined, and it was examined whether numerical changes in stoichiometric characteristics are related to climatic conditions and soil factors. In addition, the numerical change patterns of different site scaling exponents are also discussed to test whether the overall scale relationship between N and P masked significant differences in site correlation.

**Question 6:** L126-138: The study areas are not adequately described. Although it may be impossible to provide the details of all sites, the author can provide specific information about leading sites.

**Reply:**

- Thank you for your comments. It has been modified in the manuscript according to your suggestions. The research area covers almost all provinces in China (Fig 1).

Fig. 1 Sampling locations of leaf nitrogen and phosphorus for all species in this study

**Question 7:** L133: The list along with necessary details of the 413 genera and 939 species used in this study should be provided as supplementary files in order to provide clear information to the reader.

**Reply:**

- Thank you for your comments. We have provided a list along with necessary details of the 413 genera and 939 species used in this study as supplementary files according to your suggestions.

**Question 8:** L135: The list along with necessary details of the 67 sites used in this study should be provided as supplementary files to provide clear information to the reader.

**Reply:**

- Thank you for your comments. We have provided a list as supplementary files according to your suggestions.

**Question 9:** The study does not present any details regarding the research indicators measurements, which is not the appropriate method to present the study.

**Reply:**

- Thank you for your comments. It has been modified in the manuscript according to your suggestions. LN were measured using the methods described by He et al. (2006) [1]. LP was measured after H2SO4-H2O2-HF digestion via the ammonium molybdate/stannous chloride method. Stoichiometric ratios (N/P) were calculated based on dry mass [2].

**Question 10:** L153-167: The standard deviation should be added to these mean values as means + SD.

**Reply:**

- Thank you for your comments. It has been modified in the manuscript according to your suggestions.

**Question 11:** Table 1: The standard deviation should be added to these mean values as means + SD.

**Reply:**

- Thank you for your comments. It has been modified in the manuscript according to your suggestions.

**Question 12:** Figures 1, 2 and 3: Most of the results are not significant or have very low r values. Is it meaningful to present such weak or insignificant results?

**Reply:**

- Thank you for your comments. Due to the inevitable errors in data collection and aggregation, most of the results are not significant and demonstrate that environmental factors have different impacts on different life forms of woody plants. Subsequent research on the family and genus levels also confirms this result. The r value generally decreases with the increase of data volume, so although the r value is small, *P<*0.05 can indicate its significance.

**Question 13:** Figures 1, 2, 3, and 5: The sub-figures number should be added as it appears in figure 4.

**Reply:**

- Thank you for your comments. It has been modified in the manuscript according to your suggestions.

**Question 14:** 335-337: What is the reason for the inconsistent reference style?

**Reply:**

- Thank you for your comments. It has been modified in the manuscript according to your suggestions.

**Question 15:** 391: There is no information regarding the limitations of the study.

**Reply:**

- Thank you for your comments. It has been modified in the manuscript according to your suggestions. Based on the analysis of leaf N and P content data of 13 forest woody plants in eastern China, it was found that α There is a significant fluctuation, ranging from 0.57 to 1.42. Nevertheless, by integrating the N and P content data of leaves in TRY, it was found that α was the plant N and P contents follow a relatively constant power index law, as they are close to 2/3 globally and across different functional groups.

**Question 16:** 552-577: Is it necessary to include the names of all authors?

**Reply:**

- Thank you for your comments. Perhaps not necessary, just for formatting purposes. It has been modified in the manuscript according to your suggestions.

**Question 17:** At certain points in the text, it may be necessary for authors to add appropriate literature in support of the content. The following literature may be considered by authors; lines 39-42: Plant-soil interactions and C:N:P stoichiometric homeostasis of plant organs in riparian plantation (https://doi.org/10.3389/fpls.2022.979023); Effects of hydrological regime on Taxodium ascendens plant decomposition and nutrient dynamics in the Three Gorges Reservoir riparian zone (https://doi.org/10.3389/fenvs.2022.990485) and The effect of hydrological regimes on the concentrations of nonstructural carbohydrates and organic acids in the roots of Salix matsudana in the Three Gorges Reservoir, China (https://doi.org/10.1016/j.ecolind.2022.109176).

**Reply:**

- Thank you for your comments. It has been modified in the manuscript according to your suggestions.

#### **Reply to the reviewer 2**

Reviewer #2: Review summary:

This study compiled and classified a dataset of leaf nitrogen, phosphorus concentration, and nitrogen phosphorus ratio of Chinese woody plants based on different life forms. The results indicate that the internal attributes of different life forms, the growth rate related to phosphorus and the relative nutrient availability of soil might be the reasons for the unsteady relationship between nitrogen and phosphorus in leaves. Generally, this work is meaningful, but the manuscript by Duan needs to be substantially improved before being accepted for publication in Plos one at this stage. It is not yet sufficiently well written and I identify 3 major obstacles to this:

**Question 1:** There are errors in the data analysis method. Table 1 conducted an analysis of variance on tree, shrub, evergreen woody, deciduous woody and coniferous woody, which are not independent and cannot be analyzed in this way. They should be divided into three groups, including ‘trees and shrubs’, ‘deciduous forests and evergreen forest’, ‘coniferous forest and broad-leaved forests’ for independent T-test analysis. Correspondingly, the figures should also be analyzed into these three groups. The information on broad-leaved forests should be supplemented.

**Reply:**

- Thank you for your comments. It has been modified in the manuscript according to your suggestions.

| Form | n | LN | mean(mg g^−1^) | LP | mean(mg g^−1^) | N:P |  |
| --- | --- | --- | --- | --- | --- | --- | --- |
| Woody plant | 10719 | 20.77 | (8.12) | 1.58 | (1.00) | 17.28 | (22.40) |
| Tree | 5143 | 20.56 | (8.12) b | 1.56 | (1.00) a | 17.22 | (9.59) a |
| Shrub | 4970 | 21.57 | (8.06) a | 1.60 | (1.00) a | 16.92 | (8.31) a |
| Evergreen woody | 4344 | 17.43 | (6.77) b | 1.28 | (0.92) b | 18.33 | (10.32) a |
| Deciduous woody | 5772 | 23.81 | (7.95) a | 1.80 | (1.00) a | 16.11 | (7.69) b |
| Coniferous woody | 603 | 14.98 | (5.66) b | 1.54 | (0.96) a | 12.94 | (9.45) b |
| broad-leaved  woody | 10116 | 20.78 | (8.12) a | 1.58 | (1.00) a | 17.28 | (22.41) a |

**Question 2:** Some studies have shown that the N and P contents in leaves are greatly influenced by forest age. How did you consider this factor with so much data?

**Reply:**

- Thank you for your comments. Some studies have shown that the N and P contents in leaves are greatly influenced by forest age[3]. However, in most of these studies across a large scale, age sequence was not considered. Photosynthetic capacity[4] and nutrient requirements[5] generally vary with plant growth, especially for woody plants or trees. Trees at different growth stages have huge differences in physiological processes and nutrition requirements, resulting in tree nutrient stoichiometry changes along an age sequence[5, 6]. However, it remains unclear whether age variable is a determinant factor to the patterns of tree stoichiometry across a large scale. Moreover, the research data on the impact of forest age on leaf chemometrics is insufficient, leading to difficulties in big data analysis.

**Question 3:** the English language is not sufficiently well mastered so the manuscript is poorly written in English. A correction of the English language by an English-native speaker appears crucial because too many sentences and paragraphs remain incomprehensible even after revision.

**Reply:**

- Thank you for your comments. It has been modified in the manuscript according to your suggestions.

**Question 4:** Introduction: The introduction section needs further modification. The research background lacks the research progress on the stoichiometric characteristics of woody plant leaves in China. Why do you study the nitrogen and phosphorus content of woody plants in China? What is the current research progress and where is the innovation of this research?

**Reply:**

- Thank you for your comments. It has been modified in the introduction according to your suggestions. Despite extensive research on the relationship between traits in the plant kingdom, our understanding of the coordination of traits at the level of woody organisms lags behind that of herbaceous species [7]. Previous studies have identified a set of key functional traits that summarize the form and functional spectrum of the entire plant kingdom, with leaf economy and plant size being the main trait axes supporting life cycle strategies [7]. Due to the size, life span, individual development and unique structural characteristics of woody plants, compared with herbaceous plants, trees have obvious characteristics and face new abiotic stress [8]. Our current understanding of dominant trait patterns in plants fundamentally overlooks the significant energy investment structure unique to large woody species [8]. Many studies have validated some assumptions about the relationship between nitrogen, phosphorus, and environmental factors at regional and global scales [9]. However, most studies in China have not examined the different responses of leaf N, P, and N: P to environmental variables and plant growth forms [9]. This article explores the response of leaf N, P, and N: P to potential driving factors by collecting leaf nitrogen and phosphorus content data from 413 genera and 939 species of woody plants in China.

**Question 5:** Materials and methods: This section need add a distribution map of sampling points. It is recommended to organize the list of species studied and other information into the supplementary materials section. There are errors in the statistical methods. According to the previous suggestion, the analysis of variance should be changed to a T-test and divided into three groups for analysis. Additionally, information on broad-leaved forests should be supplemented.

**Reply:**

- Thank you for your comments. It has been modified in the manuscript according to your suggestions.

**Question 6:** Results: It is important to match the results reported in the table with the analysis. Also, make sure to indicate clearly if the results are significant or not. Tables and figures should be divided into three groups for analysis: ‘trees and shrubs’, ‘deciduous forests and evergreen forest’, ‘coniferous forest and broad-leaved forest’.

**Reply:**

- Thank you for your comments. It has been modified in the manuscript according to your suggestions.

**Question 7:** Discussion: Well written but the scope of the discussion does not go far enough. So far, the discussion is merely restating what other studies have already reported. The authors must offer an other aspect to their findings.

**Reply:**

- Thank you for your comments. It has been modified in the manuscript according to your suggestions.
- There are many factors that affect the stoichiometric characteristics and spatial distribution pattern of plant leaves, mainly including climate factors[10, 11], soil characteristics[9, 12], and biological factors (such as plant species, life forms, etc.)[13]. This study indicated that when the research subjects involve a large number of plant life forms, life forms often become a key driving factor for the variation of plant leaf stoichiometric characteristics, and greatly weaken the impact of climate factors and soil characteristics[1, 13, 14]. In addition, there are significant differences in the stoichiometric characteristics and spatial distribution patterns of leaves among different life forms of plants. For example, the element content in the leaves of deciduous tree species is significantly higher than that of evergreen tree species[15].

**Question 8:** Tables and figures:Table 1: It is recommended to use the form of mean ± SE for the nitrogen and phosphorus content of the data.

Figures: For figures 1 and 2, it is recommended that the format of the P-value be consistent, with some being "p<0.001" and others being "p=0.029". It is recommended to change it to "p<0.05" or "p>0.05". In addition, the P value and R2 should have the same number of decimal places retained. The colors in figures 3 and 4 should be consistent.

**Reply:**

- Thank you for your comments. It has been modified in the manuscript according to your suggestions.

Overall, you will see that we have seriously revised the manuscript based on the new results. We are confident that our manuscript has been much improved. We sincerely hope that this revised manuscript can be finally accepted for publication.

**Reference**

1.	He, J.S.; Fang, J.; Wang, Z.; Guo, D.; Flynn, D.F.; Geng, Z. Stoichiometry and large-scale patterns of leaf carbon and nitrogen in the grassland biomes of China. *Oecologia.* **2006**. *149* (1), 115-22.

2.	Sun, L.; Zhang, B.; Wang, B.; Zhang, G.; Zhang, W.; Zhang, B.; Chang, S.; Chen, T.; Liu, G. Leaf elemental stoichiometry of Tamarix Lour. species in relation to geographic, climatic, soil, and genetic components in China. *Ecological Engineering.* **2017**. *106*, 448-457.

3.	Zhang, H.; Sun, M.; Wen, Y.; Tong, R.; Wang, G.; Wu, Q.; Li, Y.; Wu, T. The effects of stand age on leaf N:P cannot be neglected: A global synthesis. *Forest Ecology and Management.* **2022**. *518*.

4.	Zheng, Y.; Zhao, Z.; Zhou, J.-J.; Zhou, H.; Liang, Z.-S.; Luo, Z.-B. The importance of slope aspect and stand age on the photosynthetic carbon fixation capacity of forest: a case study with black locust (Robinia pseudoacacia) plantations on the Loess Plateau. *Acta Physiologiae Plantarum.* **2010**. *33* (2), 419-429.

5.	Zhang, H.; Guo, W.; Yu, M.; Wang, G.G.; Wu, T. Latitudinal patterns of leaf N, P stoichiometry and nutrient resorption of Metasequoia glyptostroboides along the eastern coastline of China. *Sci Total Environ.* **2018**. *618*, 1-6.

6.	Yan, T.; Lü, X.-T.; Zhu, J.-J.; Yang, K.; Yu, L.-Z.; Gao, T. Changes in nitrogen and phosphorus cycling suggest a transition to phosphorus limitation with the stand development of larch plantations. *Plant and Soil.* **2017**. *422* (1-2), 385-396.

7.	Maynard, D.S.; Bialic-Murphy, L.; Zohner, C.M.; Averill, C.; van den Hoogen, J.; Ma, H.; Mo, L.; Smith, G.R.; Acosta, A.T.R.; Aubin, I.; Berenguer, E.; Boonman, C.C.F.; Catford, J.A.; Cerabolini, B.E.L.; Dias, A.S.; Gonzalez-Melo, A.; Hietz, P.; Lusk, C.H.; Mori, A.S.; Niinemets, U.; Pillar, V.D.; Pinho, B.X.; Rosell, J.A.; Schurr, F.M.; Sheremetev, S.N.; da Silva, A.C.; Sosinski, E.; van Bodegom, P.M.; Weiher, E.; Bonisch, G.; Kattge, J.; Crowther, T.W. Global relationships in tree functional traits. *Nat Commun.* **2022**. *13* (1), 3185.

8.	Li, J.; Chen, X.; Niklas, K.J.; Sun, J.; Wang, Z.; Zhong, Q.; Hu, D.; Cheng, D. A whole‐plant economics spectrum including bark functional traits for 59 subtropical woody plant species. *Journal of Ecology.* **2021**. *110* (1), 248-261.

9.	Chen, Y.; Han, W.; Tang, L.; Tang, Z.; Fang, J. Leaf nitrogen and phosphorus concentrations of woody plants differ in responses to climate, soil and plant growth form. *Ecography.* **2013**. *36*, 178-184.

10.	Reich, P.B.; Oleksyn, J. Global patterns of plant leaf N and P in relation to temperature and latitude. *Proceedings of the National Academy of Sciences of the United States of America.* **2004**. *101*, 11001-11006.

11.	Yuan, Z.Y.; Chen, H.Y.H. Global-scale patterns of nutrient resorption associated with latitude, temperature and precipitation. *Global Ecology and Biogeography.* **2009**. *18* (1), 11-18.

12.	Zhang, Y.; Li, C.; Wang, M. Linkages of C: N: P stoichiometry between soil and leaf and their response to climatic factors along altitudinal gradients. *Journal of Soils and Sediments.* **2018**. *19* (4), 1820-1829.

13.	Zhao, W.; Reich, P.B.; Yu, Q.; Zhao, N.; Yin, C.; Zhao, C.; Li, D.; Hu, J.; Li, T.; Yin, H.; Liu, Q. Shrub type dominates the vertical distribution of leaf C : N : P stoichiometry across an extensive altitudinal gradient. *Biogeosciences.* **2018**. *15* (7), 2033-2053.

14.	He, J.S.; Wang, L.; Flynn, D.F.; Wang, X.; Ma, W.; Fang, J. Leaf nitrogen:phosphorus stoichiometry across Chinese grassland biomes. *Oecologia.* **2008**. *155* (2), 301-10.

15.	Han, W.; Fang, J.; Guo, D.; Zhang, Y. Leaf nitrogen and phosphorus stoichiometry across 753 terrestrial plant species in China. *New Phytologist.* **2005**. *168* (2), 377-385.
