# Supplementary material for: Stoichiometric characteristics of woody plant leaves and responses to climate and soil factors in China
Source: PLoS One. 2023 Sep 21;18(9):e0291957. doi: 10.1371/journal.pone.0291957 (PMC10513206; doi:10.1371/journal.pone.0291957)
Supplement: Attachment — Submitted filename: Reply to the editor and reviewers20230525.doc [file pone.0291957.s004.doc]

#### **Reply to the editor and reviewers**

#### **Manuscript ID**: PONE-D-23-11129

**Title:**	Stoichiometric characteristics of woody plant leaves and responses to climate and soil factors in China

**Authors:** Xiangguang Duan

Dear Ph.D. Guo,

First of all, I should thank you and the reviewers for your expert comments and valuable suggestions to our manuscript. After reading the comments, I came to realize that there were indeed some weak points and unsuitable expression in our original manuscript. The issues raised by you and the two reviewers were of great help for our revision.

In the past few days, I have seriously revised the original manuscript. As you will see, all the questions and suggestions were taken into consideration in the revised manuscript. Below I explain in detail how the questions and suggestions were addressed.

#### **Reply to the reviewer 2**

Reviewer #1: Review summary:

While revising, the author followed almost all of the comments and made significant progress.

Reviewer #2: Review summary:

There are still many issues with the manuscript that need further revision, especially with regards to the figures and tables.

**Question 1:** Figure 1: The map scale is out of balance and there are too many blank spaces. Please adjust it.

**Reply:**

- Thank you for your comments. It has been modified according to your suggestions.

Fig. 1 Chinese sampling locations of leaf nitrogen and phosphorus levels for all species in this study

**Question 2:** L194: Table1 “Different letters (such as a, b, and c) represent significant differences (p < 0.05)” There is no ‘c’ in the significance difference

**Reply:**

- Thank you for your comments. It has been modified according to your suggestions.
- Table 1. Summary of the statistics of N and P concentrations and N: P ratios in leaves of different functional groups of terrestrial plants. Multiple comparisons were made for each group of trees and shrubs, evergreen and deciduous, coniferous and broad-leaved. Mean represents the geometric mean, and n represents the number of observations. Different letters (such as a and b) represent significant differences (*p* < 0.05).

**Question 3:** Table 1: The T-test did not indicate in the table which two groups were between, and the representation was incorrect, so it should not be represented in a single row

**Reply:**

- Thank you for your comments. It has been modified according to your suggestions.
- Table 1. Summary of the statistics of N and P concentrations and N: P ratios in leaves of different functional groups of terrestrial plants. Multiple comparisons were made for each group of trees and shrubs, evergreen and deciduous, coniferous and broad-leaved. Mean represents the geometric mean, and n represents the number of observations. Different letters (such as a and b) represent significant differences (p < 0.05).

| Form | n | LN | mean(mg g^−1^) | LP | mean(mg g^−1^) | N:P |  |
| --- | --- | --- | --- | --- | --- | --- | --- |
| Woody plant | 10719 | 20.77 | ±8.12 | 1.58 | ±1.00 | 17.28 | ±22.40 |
| Tree | 5143 | 20.56 | ±8.12 b | 1.56 | ±1.00 a | 17.22 | ±9.59 a |
| Shrub | 4970 | 21.57 | ±8.06 a | 1.60 | ±1.00 a | 16.92 | ±8.31 a |
| Evergreen woody | 4344 | 17.43 | ±6.77 b | 1.28 | ±0.92 b | 18.33 | ±10.32 a |
| Deciduous woody | 5772 | 23.81 | ±7.95 a | 1.80 | ±1.00 a | 16.11 | ±7.69 b |
| Coniferous woody | 603 | 14.98 | ±5.66 b | 1.54 | ±0.96 a | 12.94 | ±9.45 b |
| Broad-leaved  woody | 10116 | 20.78 | ±8.12 a | 1.58 | ±1.00 a | 17.28 | ±22.41 a |

**Question 4:** Table 1: I don't understand what the numbers in parentheses represent, they should be expressed as mean±SE.

**Reply:**

- Thank you for your comments. It has been modified in the manuscript according to your suggestions.
- Table 1. Summary of the statistics of N and P concentrations and N: P ratios in leaves of different functional groups of terrestrial plants. Multiple comparisons were made for each group of trees and shrubs, evergreen and deciduous, coniferous and broad-leaved. Mean represents the geometric mean, and n represents the number of observations. Different letters (such as a and b) represent significant differences (p < 0.05).

| Form | n | LN | mean(mg g^−1^) | LP | mean(mg g^−1^) | N:P |  |
| --- | --- | --- | --- | --- | --- | --- | --- |
| Woody plant | 10719 | 20.77 | ±8.12 | 1.58 | ±1.00 | 17.28 | ±22.40 |
| Tree | 5143 | 20.56 | ±8.12 b | 1.56 | ±1.00 a | 17.22 | ±9.59 a |
| Shrub | 4970 | 21.57 | ±8.06 a | 1.60 | ±1.00 a | 16.92 | ±8.31 a |
| Evergreen woody | 4344 | 17.43 | ±6.77 b | 1.28 | ±0.92 b | 18.33 | ±10.32 a |
| Deciduous woody | 5772 | 23.81 | ±7.95 a | 1.80 | ±1.00 a | 16.11 | ±7.69 b |
| Coniferous woody | 603 | 14.98 | ±5.66 b | 1.54 | ±0.96 a | 12.94 | ±9.45 b |
| Broad-leaved  woody | 10116 | 20.78 | ±8.12 a | 1.58 | ±1.00 a | 17.28 | ±22.41 a |

**Question 5:** Figure 3: Why is the comparison of broad-leaved tree species not included in the figure?

**Reply:** Thank you for your comments. It has been modified in the manuscript according to your suggestions.

Fig. 2 Relationship between stoichiometry of leaves of different life forms and climatic factors

Fig. 3 Relationship between stoichiometry of leaves of different life forms and soil factors

**Question 6:** All fonts in the figures should be consistent, such as inconsistent fonts in Figures 4 and 5.

**Reply:**

- Thank you for your comments. It has been modified in the manuscript according to your suggestions.

Fig. 4 Relationship between N-P scale index and MAT, MAP, soil AN, and soil AP at different sites. Climate and soil values are obtained from the geometric average of each station. The proportion index was calculated through SMA regression of leaf nitrogen and phosphorus concentration (α), such as log_10_LN = α log_10_LP + log_10_ β.

Fig. 5 Relationship between N-P ratio index and N, P, and N: P mass ratio of leaves at different sites. (a) Relationship between nitrogen and phosphorus ratio index and nitrogen content of leaves; (b) relationship between N: P ratio index and P content in leaves; (c) relationship between N-P proportional index and N: P mass ratio. Leaf N and P contents and N: P ratio are calculated from the geometric mean value of each station. The proportion index was calculated through SMA regression of leaf N and P concentration (α). For example, log_10_LN = α log_10_LP + log_10_ β.

**Question 7:** The serial numbers of each small image in Figure 5 are in lowercase letters, while in other images, uppercase letters are used. Please unify them

**Reply:**

- Thank you for your comments. It has been modified in the manuscript according to your suggestions.
-

Fig. 5 Relationship between N-P ratio index and N, P, and N: P mass ratio of leaves at different sites. (a) Relationship between nitrogen and phosphorus ratio index and nitrogen content of leaves; (b) relationship between N: P ratio index and P content in leaves; (c) relationship between N-P proportional index and N: P mass ratio. Leaf N and P contents and N: P ratio are calculated from the geometric mean value of each station. The proportion index was calculated through SMA regression of leaf N and P concentration (α). For example, log10LN = α log10LP + log10 β.

**Question 8:** Discussion: L316: Please indicate which one is in Figure 4A, B, C or D? The same problem also exists in L337, 357…….

**Reply:**

- Thank you for your comments. It has been modified in the manuscript according to your suggestions.
- This value also varies among different sites and is significantly correlated with climatic factors, while soil factors have no significant impact on the scaling exponent (Fig. 4A, 4C and 4D).
- The N-P scaling exponent was mainly determined by life form and MAT (Table 2, Fig. 4A).
- Compared with LN, LP changed more and was also more closely related to climate and soil (Table 1, Fig. 6A and 6B)
- LN was mainly determined by the changes of plant growth morphology along the latitude gradient, while leaf P and N: P were determined by both MAT and plant growth morphology (Fig. 6A, 6B and 6C).
- Correlation analysis of the N-P scaling exponent with climatic and soil factors showed that it was only significantly negatively correlated with MAT, but not with soil factors (Fig. 4A, 4Cand 4D).

Overall, you will see that we have seriously revised the manuscript based on the new results. We are confident that our manuscript has been much improved. We sincerely hope that this revised manuscript can be finally accepted for publication.
